# Tackling COVID‐19 infection through complement‐targeted immunotherapy

**DOI:** 10.1111/bph.15187

**Published:** 2020-07-27

**Authors:** Sonata Jodele, Jörg Köhl

**Affiliations:** ^1^ Division of Bone Marrow Transplantation and Immune Deficiency, Cancer and Blood Disease Institute Cincinnati Children's Hospital Medical Center, University of Cincinnati College of Medicine Cincinnati Ohio USA; ^2^ Department of Pediatrics University of Cincinnati College of Medicine Cincinnati Ohio USA; ^3^ Division of Immunobiology Cincinnati Children's Hospital Medical Center, University of Cincinnati College of Medicine Cincinnati Ohio USA; ^4^ Institute for Systemic Inflammation Research University of Lübeck Lübeck Germany

## Abstract

The complement system is an ancient part of innate immunity sensing highly pathogenic coronaviruses by mannan‐binding lectin (MBL) resulting in lectin pathway activation and subsequent generation of the anaphylatoxins (ATs) C3a and C5a as important effector molecules. Complement deposition on endothelial cells and high blood C5a serum levels have been reported in COVID‐19 patients with severe illness, suggesting vigorous complement activation leading to systemic thrombotic microangiopathy (TMA). Complement regulator gene variants prevalent in African‐Americans have been associated with a higher risk for severe TMA and multi‐organ injury. Strikingly, severe acute respiratory syndrome Coronavirus 2 (SARS‐CoV‐2)‐infected African‐Americans suffer from high mortality. These findings allow us to apply our knowledge from other complement‐mediated diseases to COVID‐19 infection to better understand severe disease pathogenesis. Here, we discuss the multiple aspects of complement activation, regulation, crosstalk with other parts of the immune system, and the options to target complement in COVID‐19 patients to halt disease progression and death.

**Linked Articles:**

This article is part of a themed issue on Canonical and non‐canonical functions of the complement system in health and disease. To view the other articles in this section visit http://onlinelibrary.wiley.com/doi/10.1111/bph.v178.14/issuetoc

**Abbreviations**
aHUS: atypical haemolytic uraemic syndrome
ALI: acute lung injury
AMD: age‐related macular degeneration
AP: alternative pathway
ARDS: acute respiratory distress syndrome
AT: anaphylatoxin
BAL: bronchoalveolar lavage fluid
C1INH: C1 esterase inhibitor
C4BP: C4 binding protein
CAD: cold agglutinin disease
CoV: Coronavirus
CP: classical pathway
CR: complement receptor
DAF: decay accelerating factor
DC: dendritic cell
FB: factor B
FD: factor D
FH: factor H
FHR: factor H‐related
FI: factor I
FP: properdin
HLH: haemophagocytic lymphohistiocytosis
HSCT: haematopoietic stem cell transplantation
LP: lectin pathway
MAC: membrane attack complex
MASP: mannan‐binding lectin serine protease
MBL: mannan‐binding lectin
MCP: membrane cofactor protein
N protein: nucleocapsid protein
NET: neutrophil extracellular trap
PNH: paroxysmal nocturnal haemoglobinuria
RCA: regulator of complement activation
S protein: spike protein
SARS: severe acute respiratory syndrome
sC5b‐9: soluble C5b‐9
SNP: single nucleotide polymorphism
TA‐TMA: transplant‐associated TMA
TMA: thrombotic microangiopathy

## 1 INTRODUCTION

COVID‐19 infection by severe acute respiratory syndrome (SARS) Coronavirus (CoV) 2 was first identified in December of 2019 in China. Since then, it has rapidly spread worldwide and caused a pandemic that unites clinicians and researchers around the globe in their efforts to understand disease mechanisms and the host response, in order to design effective clinical interventions. The United States is one of the countries with highest incidence of COVID‐19 with 1,839,1674 cases diagnosed and 106,312 total deaths by June 3, 2020 (https://gisanddata.maps.arcgis.com/apps/opsdashboard/index.html#/bda7594740fd40299423467b48e9ecf6).

SARS‐CoV‐2 belongs to the family of Coronaviridae, positive‐sense single stranded RNA viruses that frequently cause mild respiratory infections in humans. During the past two decades, two endemics with SARS‐CoV in 2003 and Middle East respiratory syndrome (MERS)‐CoV in 2012 occurred with estimated case fatalities of 14–15% or even 35% in case of MERS‐CoV (Gao et al., 2020). COVID‐19 disease has a wide range of clinical presentations from asymptomatic cases to severe respiratory involvement acutely progressing to acute respiratory distress syndrome (ARDS) and multi‐organ failure. A growing body of literature on COVID‐19 reports atypical presentation of ARDS (Gattinoni et al., 2020), as a result of host immune system overactivation and fatal hypercytokinaemia, leading to tissue injury and multi‐organ failure, which may be attributed to an overactivated complement system (Gao et al., 2020; Lipworth, Chan, Lipworth, & RuiWen Kuo, 2020; Magro et al., 2020; Risitano et al., 2020).

## 2 COMPLEMENT ACTIVATION AND FUNCTION IN HIGHLY PATHOGENIC CORONAVIRUS INFECTIONS

**FIGURE 1:**
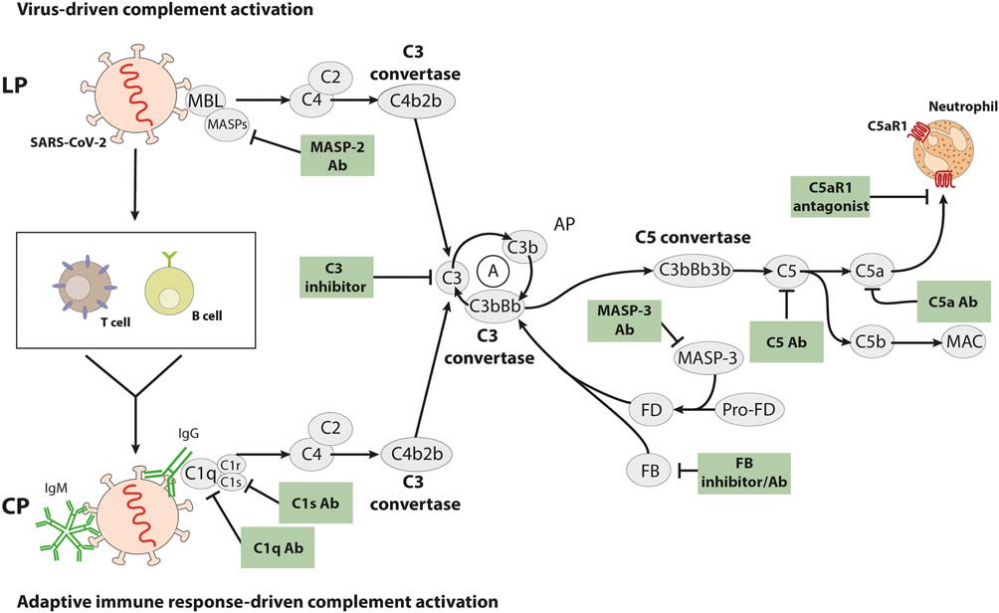
SARS‐CoV‐2‐driven complement activation and potential targets of the complement cascade. Several structural proteins of SARS‐CoV‐2 including the S and N proteins are recognized by MBL resulting in virus‐induced activation of the LP. Sensing of the virus by the innate immune system leads to the activation of B and T cells of the adaptive immune system and the production of virus‐specific IgM and IgG antibodies. Such antibodies can activate the complement system by the CP. LP and CP activation initiate a cascade of proteolytic events resulting in the assembly of the C3 convertase C4b2b, eventually cleaving C3 into C3a and C3b. This C3b serves as the nucleus of the amplification (A) loop, which drives the ongoing cleavage of C3 unless controlled by complement regulator proteins. The emerging C3 convertase of the AP, C3bBb, can form the C5 convertase C3bBb3b of the AP, which cleaves C5 into C5a and C5b. C5 serves as the nucleus for MAC/C5b‐9 formation. Several compounds have been generated that specifically target the activation of the LP at the level of the serine protease MASP‐2 or the CP at the level of C1q and C1s. Further, compounds have been developed to inhibit the cleavage of C3 by either targeting molecules that build the AP C3 convertase or by protecting C3 from C3 convertase‐mediated cleavage. Downstream of C3, antibodies and inhibitors of C5 have been generated that protect C5 from cleavage by the C5 convertases. Finally, antibodies or other molecules have been developed that target either C5a or its primary receptor, C5aR1. The potential use of the different complement inhibitors in COVID‐19 infection is discussed in the text

The complement system senses invading pathogens as well as environmental or self‐derived antigens by pattern recognition molecules of the canonical classical and lectin pathways (CP and LP). This function is critical to our sustained health and survival. It is characterized by a cascade of proteolytic events leading to the cleavage of C3 into the anaphylatoxin (AT) C3a and the opsonin C3b by pathway‐specific canonical C3 convertases. Consecutively, such C3 convertases build the framework for C5 convertases that cleave C5 into the AT C5a and C5b. In addition to CP and LP activation, the thioester in C3 can be directly activated by any nucleophilic attack leading to the activation of the so‐called alternative pathway (AP), driving the generation of substantial amounts of C3a and C5a. Eventually, C5b serves as the nucleus of non‐proteolytic terminal pathway activation leading to the formation of the soluble (s)C5b‐9 complex in the circulation and the pore‐forming membrane attack complex (MAC) on cell surfaces, which can directly lyse cells (Figure 1).

Similar to SARS‐CoV, the genome of SARS‐CoV‐2 encodes for several structural and non‐structural proteins including the spike (S) protein, which is critical for cell entry through engagement of ACE2 and the employment of the cellular serine protease TMPRSS2 for spike protein (S protein) priming (Hoffmann et al., 2020). The S protein of SARS‐CoV is sensed by mannan‐binding lectin (MBL) suggesting that complement activation in SARS‐CoV infection is driven by activation of the LP (Ip et al., 2005; Zhou et al., 2010). Also, nucleocapsid (N) protein interaction with mannan‐binding lectin serine protease (MASP)‐2, the key protease of LP activation, has been described for SARS‐CoV, MERS‐CoV, and SARS‐CoV‐2 and affects LP activation (Figure 1) (Gao et al., 2020). In addition to MBL/MASP‐2‐driven activation of the LP, complement might be activated by the CP through virus‐neutralizing IgG antibodies (Figure 1). In COVID‐19 patients, seroconversion occurred at a similar time or slightly earlier than was seen in SARS‐CoV patients. Around 50% of COVID‐19 patient showed seroconversion on day 7 after development of symptoms (Wolfel et al., 2020). Of note, in SARS‐CoV‐infected patients, the appearance of anti‐viral IgG coincided with the onset of ARDS in 80% of patients (Peiris et al., 2003).

Complement activation by the three activation pathways results in the generation of the small cleavage fragments of C3 and C5, the ATs C3a and C5a. They are important effector molecules that attract, activate, and regulate innate and adaptive immune cells (Laumonnier, Karsten, & Kohl, 2017). C5a exerts powerful proinflammatory properties through activation of such proinflammatory cells. For example, C5a induces the expression of IL‐1β and CXCL8/IL‐8 in mononuclear cells and enhances the release of IL‐6 and TNF‐α (Schindler, Gelfand, & Dinarello, 1990).

The development of ARDS is mediated by the recruitment and activation of inflammatory cells such as neutrophils, eosinophils, monocytes, and T lymphocytes (Meliopoulos et al., 2014). Similar to SARS‐CoV‐2, MERS‐CoV, or SARS‐CoV infection, influenza virus infection can be associated with a rapid progression to ARDS. MERS‐CoV drives the production of inflammatory and chemotactic cytokines as well as chemokines such as CXCL‐10, CCL2, IL‐8, IL‐12, and IFN‐γ, which can cause severe lung damage (Jiang et al., 2018, 2019). High levels of C5a have been found in bronchoalveolar lavage fluid (BAL) of individuals affected by viral‐mediated acute lung injury (ALI) but not in BAL from recovered patients with ARDS (Wang, Xiao, Guo, Li, & Shen, 2015). Importantly, the histopathological changes in the lung from patients infected with influenza virus mimic those infected with SARS‐CoV (Meliopoulos et al., 2014). The influenza virus is highly pathogenic and replicates in the lower respiratory tract. It drives pulmonary complement activation leading to high levels of C5a in BAL and serum (Ohta et al., 2011). In experimental, highly pathogenic avian influenza H5N1 infection, C5a contributes to ALI. Further, inhibition of C5a by a C5a‐specific mAb alleviated such lung injury in H5N1 virus infection in this mouse model (Sun et al., 2013). Similarly, anti‐C5a mAb treatment improved the outcome of H7N9 virus infection in African green monkeys; in particular, such treatment attenuated ALI and systemic inflammation, that is, the “cytokine storm” (Sun et al., 2015). Perhaps of even more importance, blockade of the C5a/C5aR1 axis reduced lung and spleen tissue damage and the inflammatory response in experimental MERS‐CoV infection. Also, C5a/C5aR1 blockade decreased viral replication in the lung. Recently, it was further shown that C3‐deficient mice infected with SARS‐CoV suffered from less respiratory dysfunction associated with less recruitment of neutrophils and inflammatory monocytes in the lungs and lower cytokine and chemokine levels (Gralinski et al., 2018).

Taken together, the available data suggest that complement is activated in highly pathogenic coronavirus infections and contributes to the development of ALI that has been observed in experimental models and in patients. In the following sections, we will discuss complement‐mediated microvascular injury in COVID‐19 patients, complement genetics as a potential clue to race differences in COVID‐19 severity, options to target complement in COVID‐19 patients with atypical ARDS and thrombotic microangiopathy (TMA), and potential interaction of complement with other inflammatory pathways, offering the opportunity for concurrent interventions.

## 3 COMPLEMENT‐ASSOCIATED MICROVASCULAR INJURY IN SEVERELY ILL COVID‐19 PATIENTS

It is currently unknown why some patients with SARS‐CoV‐2 infection develop mild symptoms while others progress to severe COVID‐19 illness and multisystem organ failure with high mortality rates. It is also unknown why some patient populations, especially African‐Americans, are at a higher risk of developing severe complications in response to SARS‐CoV‐2 infection. Recent autopsy data from New Orleans in four African‐American patients who succumbed to COVID‐19 infection demonstrated diffuse alveolar damage and TMA associated with foci of alveolar haemorrhage in the lungs (Fox et al., 2020). One of the cases had extensive fibrin with degenerated neutrophils within the alveoli, possibly representing neutrophil extracellular traps (NETs). RNA imaging in available samples showed multinucleated cells within alveolar spaces with abundant RNA, likely to represent virally infected cells, as had been previously reported in a post‐mortem case from China (Xu et al., 2020). The most significant gross cardiac finding was cardiomegaly with right ventricular dilatation in all patients without evidence of myocarditis. Elevated B‐type natriuretic peptide associated with right ventricular dilatation was documented at least in one case. The authors considered these findings consistent with recent observations by Chen et al. who hypothesized that pericytes may be infected by the SARS‐CoV‐2 virus and cause capillary endothelial cell microvascular dysfunction eventually causing individual cardiac cell necrosis (Chen, Li, Chen, Feng, & Xiong, 2020). There were no documented secondary bacterial or fungal infections, although all patients received antimicrobial treatments during critical illness. Based on these findings, the authors concluded that effective therapy for these patients should include targeted therapy for TMA in addition to virus‐directed therapies. Pulmonary abnormalities in severely affected patients are largely restricted to the alveolar capillaries, presenting as TMA with some evidence of viral cytopathic changes in alveolar lining. It is known that the virus uses the ACE2 receptor expressed by pneumocytes in the epithelial alveolar lining to infect the host and causing lung injury. However, since ACE2 receptors are also widely expressed on vascular endothelial cells, many other organs can be affected (Ou et al., 2020).

**FIGURE 2:**
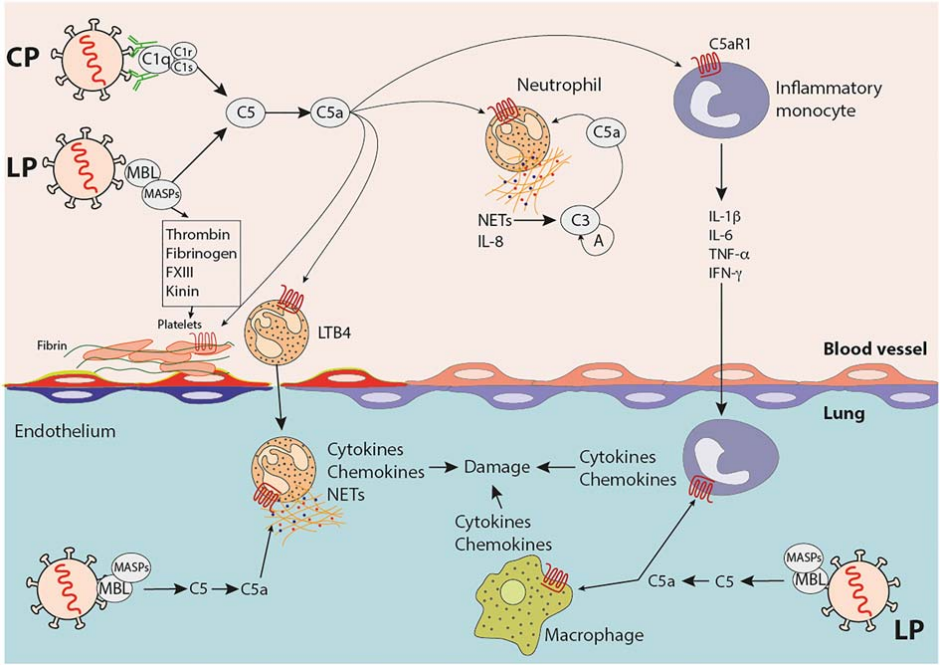
Complement effector functions leading to SARS‐CoV‐2‐induced thrombotic microangiopathy (TMA) and acute lung injury (ALI). SARS‐CoV‐2 sensing by pattern recognition molecules of the LP and CP results in C5 cleavage and the generation of the AT C5a. Further, LP‐derived MAPSs activate the coagulation and the kinin system to drive TMA development leading to fibrin formation and platelet aggregation. C5a attracts neutrophils and inflammatory monocytes to adhere to the vascular endothelium, release IL‐8 and multiple inflammatory cytokines, and form NETs. Such NETs can activate complement by the AP and fuel the C3 amplification loop (A), when complement regulators are exhausted and/or when their function is reduced due to loss‐of‐function mutations. Also, adherent neutrophils produce LTB_4_ that binds to and activates its cognate receptor. Consequently, neutrophils transmigrate into the lung. C5a‐activated monocytes in concert with activated neutrophils produce proinflammatory cytokines and chemokines that further activate the endothelium and amplify inflammation. Virus‐induced complement activation by the LP within the lung tissue serves as an additional source of C5a. Such C5a activates neutrophils and inflammatory monocytes that were recruited to the lung as well as tissue‐resident macrophages to produce proinflammatory chemokines and cytokines, eventually driving tissue damage leading to ALI and ARDS

Recent studies showed complement‐associated microvascular injury and thrombosis in critically ill SARS‐CoV‐2‐infected patients. These studies documented extensive deposits of the terminal complement complex C5b‐9, C4d, and MASP‐2 in small vessels of affected organs (Fox et al., 2020; Magro et al., 2020). Co‐localization of complement components C5b‐9 and C4 with SARS‐CoV‐2 S protein indicated viral invasion of vascular endothelial cells, which also had been demonstrated by electron microscopy showing viral inclusion structures in vascular endothelial cells in lungs, heart, kidney, gastrointestinal tract, and the skin (Magro et al., 2020). A recent study from Wuhan identified strong staining for MBL, MASP‐2, C4a, C3b, and C5b‐9 in type I and type II alveolar epithelial cells, inflammatory cells, pneumocytes, and even in exudates in alveolar spaces with necrotic cell debris (Gao et al., 2020). There is potential interaction of complement with coagulation pathways resulting in acutely progressive microthrombosis with fibrin deposition and highly elevated D‐dimers (Figure 2) (Ekdahl et al., 2016). Regardless of the initial insult leading to TMA, complement‐mediated vascular endothelial injury may respond to complement‐modulating therapies and offers the opportunity to adopt insights regarding disease mechanisms and therapeutic interventions from TMAs of other origins to COVID‐19.

## 4 COMPLEMENT GENETICS AS A POTENTIAL CLUE TO RACE DIFFERENCES IN COVID‐19 SEVERITY

**FIGURE 3:**
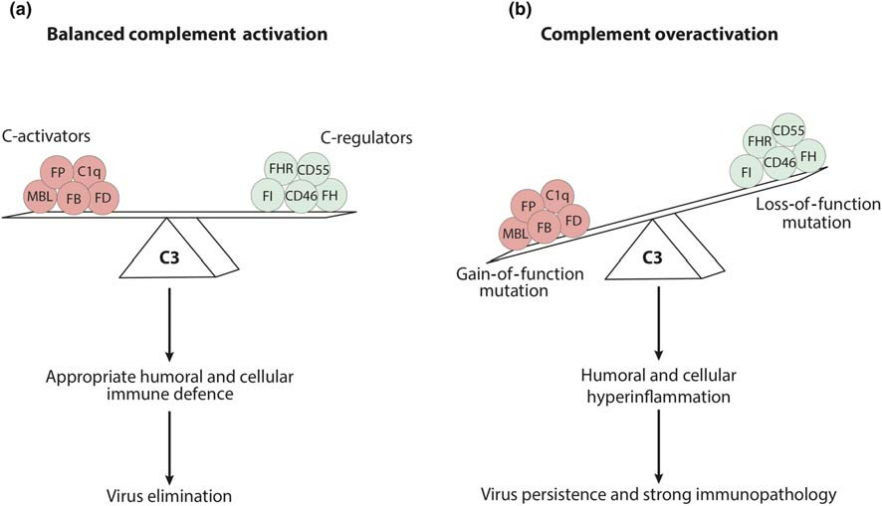
Effects of inherited gain‐ or loss‐of‐function mutations in complement activator or regulator proteins on complement pathway activation. The degree of complement activation in response to infection is defined by the strength of the activation by complement activator molecules, the formation of the critical AP amplification loop, and the potency of the system to balance this activation by complement regulator molecules. (a) Balanced complement activation occurs, when sensing of the SARS‐CoV‐2 virus by MBL or virus‐specific antibodies is appropriately controlled by complement regulator proteins resulting in innate immunity‐guided activation of adaptive immune responses, eventually leading to virus elimination. (b) Polymorphisms in a set of complement proteins, either associated with a gain of function in complement activators or loss of function in complement regulators, or both, can aggregate to effects leading to complement overactivation as has been observed in African‐Americans with HSCT‐TMA. In case of SARS‐CoV‐2 infection, such aggregation of inherited variants of complement proteins may lead to humoral and cellular hyperinflammation, associated with virus persistence and strong immunopathology causing TMA, ALI, and/or ARDS

One of the current uncertainties in COVID‐19 infection is the racial difference in clinical presentations in patients developing severe illness. As outlined above, highly pathogenic coronaviruses are recognized by MBL. Several polymorphisms have been described for MBL in exon 1 at codon positions 52, 54, and 57 (Steffensen, Thiel, Varming, Jersild, & Jensenius, 2000). The A allele can be distinguished from R52C, G54D, and G57E polymorphisms described as D, B, and C alleles (Garred, Larsen, Seyfarth, Fujita, & Madsen, 2006). These polymorphisms in exon 1 together with those in the promoter region profoundly affect circulating levels of MBL (Madsen et al., 1995). Importantly, MBL polymorphisms have been associated with fatal outcome in patients with sepsis, SIRS (Garred, Strøm, Quist, Taaning, & Madsen, 2003; Hellemann et al., 2007), and ARDS (Gong et al., 2007). Also, some (Ip et al., 2005; Tu et al., 2015; Zhang et al., 2005) but not all (Yuan et al., 2005) studies found a significant association between MBL codon variants in exon 1 and the risk of severe SARS‐CoV infection. Thus, polymorphisms in exon 1 and/or the promoter region of MBL may define the extent of complement activation in COVID‐19 patients (Figure 3). In support of this view, strong differences have been observed between haplotype frequencies in Asians, Caucasians, Hispanic, and African‐Americans (Garred et al., 2006). Intriguingly, the G54D polymorphism is extremely rare in West Africa but can be found at higher frequencies in Caucasians, Asians, and indigenous South Americans respectively. In contrast, the C allele is more frequent in sub‐Saharan Africa but rare among Caucasians. The D allele is largely restricted to North Africans and Caucasians. It has been suggested that environmental pressures such as tuberculosis infections could account for the fact that almost 60% of the sub‐Saharan population contains the C allele (Bernig et al., 2004). In support of this possibility, a protective association has been described for the C allele and tuberculosis infection with *Mycobacterium africanum* (Thye et al., 2011). Thus, racial differences in MBL‐mediated virus sensing may lead to different complement activation in COVID‐19.

In addition to MBL, complement genetics studies in haematopoietic stem cell transplantation (HSCT) recipients provide evidence for racial disparities in predisposition to develop TMA and risk of mortality in response to transplantation. These findings may explain the higher mortality seen in African‐Americans with COVID‐19 infection. A previous large HSCT registry report described inferior survival in African‐Americans after unrelated donor HSCT, but it was unable to attribute reduced outcomes to risk factors such as HLA‐matching and socio‐economic status (Baker et al., 2009). A prospective study examining genetic predisposition for transplant‐associated thrombotic microangiopathy (TA‐TMA) in HSCT recipients demonstrated that 65% of patients with TA‐TMA had genetic variants in at least one complement gene as compared with 9% of patients without TA‐TMA (*P* < 0.0001) using a hypothesis‐driven 17‐gene panel including the complement factors *C3*, *factor B (FB)* , *C5*, *FP*, *FD* , *FI*, *FH*, *FH‐related (FHR)1*, *FHR3*, *FHR4*, *FHR5*, *decay accelerating factor (DAF/CD55)*, *CD59*, *membrane cofactor protein (MCP/CD46)*, and *C4BPA* (Jodele et al., 2016). Importantly, many of the complement genes belong to complement regulators that either control the amplification of the cascade at the level of C3, that is, FH, CD55, CD46, and FI, or fuel the amplification loop (FB, FP, and FD).

Complement gene variants were increased in patients of all races with TA‐TMA, but African‐Americans had more variants than Caucasians. While the *FD* variant (c.357116C.A) was detected only in African‐American patients with TMA, it was mainly the number of variants occurring in individuals with TA‐TMA and not a particular gene variant that was significantly associated with TMA and disease severity. Variants in ≥3 genes were identified only in African‐Americans with TA‐TMA and were associated with very high mortality after HSCT (71%) and strong complement activation (Jodele et al., 2014). The finding of multiple variants occurring at high frequency in persons of African descent associated with strong complement activation suggests a selective benefit of strong complement activation in Africans as a defence mechanism to combat pathogens like *Neisseria meningitides*, a prevalent cause of mortality in endemic areas of Africa (African meningitis belt). Notably, single nucleotide polymorphism (SNPs) in C3 (Adriani et al., 2013) and FH have been associated with susceptibility (Davila et al., 2010) to this pathogen.

The homeostasis of complement is controlled by an equilibrium between activation and control. Dysregulation of complement activation at the level of C3 through loss‐ or gain‐of‐function mutations of regulators or gain‐of‐function mutations in activator proteins results in uncontrolled complement activation and inflammation as seen in many inflammatory conditions including HSCT‐TMA (Figure 3). The combination of several complement gene polymorphisms, in particular, in *C3*, *FH*, and *FB*, has been found to determine systemic complement activity and the susceptibility to AP‐driven diseases (Heurich et al., 2011; Paun et al., 2016). Thus, the available data from HSCT‐TMA suggest that African‐Americans with multiple variations in complement genes react with stronger complement activation in response to infection with highly pathogenic coronaviruses including SARS‐COV‐2, resulting in uncontrolled pulmonary tissue inflammation and complement deposition in several organs.

In support of this view, we found at least one complement gene variant in 32% of 50 African‐American and 50 Caucasian children. The frequency of gene variants differed markedly by race with 50% of African‐American children having at least one gene variant, compared to only 14% of Caucasian children although the overall variant frequencies were somewhat lower than those reported in HSCT recipients (Jodele et al., 2017).

Based on these observations, it will be important to examine genetic variants of complement proteins in patients infected with SARS‐CoV‐2 and to correlate such variants with disease severity. Such data might help to predict the risk of developing virus‐associated TMA.

## 5 COMPLEMENT THERAPY IN COVID‐19 PATIENTS

Many teams around the world are striving to identify effective therapies for COVID‐19. SARS‐CoV‐2 preventive measures aim to develop an effective vaccine. Potential therapeutic approaches include different strategies of anti‐viral medications and virus‐specific T cells that have high potential for being effective in the future. The clinical appearance of severely ill COVID‐19 patients with atypical ARDS and TMA and the association of this clinical phenotype with marked complement activation in the circulation and in the lung (Gao et al., 2020; Lipworth et al., 2020; Magro et al., 2020) suggests that complement may also serve as a target in COVID‐19 patients. Encouraging preliminary data have been reported for severely ill COVID‐19 subjects, who were either treated with a C3 antagonist (Mastaglio et al., 2020) and C5‐ or C5a‐blocking antibodies (Diurno et al., 2020; Gao et al., 2020). These approaches resulted in rapid clinical improvement. In this section, we will discuss strategies to target complement at different levels. In particular, we will take into account clinical experience obtained with C5 targeting in different complement‐mediated TMAs to prevent acute mortality from atypical ARDS and/or multiple organ failure in COVID‐19‐affected individuals.

### 5.1 Complement pathways as potential targets

The available data suggest that the LP and the CP are the two major complement pathways activated in response to SARS‐CoV‐2. MBL, collectins 10 and 11, and the ficolins 1–3 function as soluble sensors of the LP (Garred et al., 2016). They interact with MASP‐1 and MASP‐2 that act in concert to cleave C4 and C2 and form the C3 convertase C4b2b, which proteolytically cleaves C3 into C3a and C3b (Figure 1). MBL comprises multiple carbohydrate recognition domains that can bind to the high‐mannose structure of the SARS‐CoV S protein (Ip et al., 2005). In particular, the N‐linked glycosylation site N330 on the S protein seems to be critical for MBL interaction. Further, MBL directly inhibited SARS‐CoV‐mediated infection in vitro (Zhou et al., 2010). In addition to the S protein, a recent report uncovered the interaction of LP MASP‐2 with a highly conserved motif in the nucleocapsid protein (N protein) of SARS‐CoV, SARS‐CoV‐2, and MERS‐CoV. Intriguingly, this interaction not only potentiated MASP‐2‐driven activation of the LP but also aggravated LPS‐induced pneumonia in a MASP‐2‐dependent way (Gao et al., 2020). Given that the N protein is one of the most abundant structural proteins in the serum of patients infected by SARS‐CoV (Che et al., 2004; Chen et al., 2005; Guan et al., 2004), its interaction with MASP‐2 could serve as an important amplifier of LP activation in highly pathogenic coronavirus infection. Thus, the available data point towards the LP as an important target in these virus infections.

C1q is the sensor molecule of the CP that recognizes multiple conserved molecular patterns including IgM or IgG hexamer molecules that have bound their cognate antigen (Diebolder et al., 2014). Similar to MASP‐1 and MASP‐2, the serine proteases C1r and C1s form a complex with IgG/IgM‐bound C1q to cleave C4 and C2 and generate the C4b2b convertase, eventually cleaving C3 into C3a and C3b (Figure 1). Within the first week after symptoms, COVID‐19 infection results in the production of S protein‐specific IgG/IgM antibodies as a target structure for C1q (Wolfel et al., 2020). A recent study from Wuhan shows that during the first 5 days after clinical onset, already 30–40% of the infected individuals have generated IgM or IgG antibodies directed against the S or N proteins of SARS‐CoV‐2, with a slightly higher frequency of antibodies against the S protein (Liu et al., 2020). After 5 weeks, all of the 214 tested patients showed IgG seroconversion. These data suggest that the seroconversion is somewhat quicker than that observed with SARS‐CoV (Peiris et al., 2003). Taken together, CP activation by IgM and IgG antibodies directed against the S and N proteins of SARS‐CoV and SARS‐CoV‐2 serves as second mechanism of complement activation in addition to the initial virus sensing by the MBL and LP activation (Figure 1).

The C3 convertase C4b2b, which is assembled in response to LP and CP activation, generates the AT C3a from C3, which can degranulate basophils and mast cells leading to histamine release through activation of its cognate C3aR (El‐Lati, Dahinden, & Church, 1994; Kretzschmar et al., 1993). C3aR expression is triggered in neutrophils upon LPS exposure and contributes to NETosis (Guglietta et al., 2016). Further, C3a induces aggregation and 5‐HT (serotonin) release from platelets, regulates secretion of IL‐6 and TNF‐α from B cells and monocytes, and leads to the production of IL‐8 by an epithelial cell line (Fischer & Hugli, 1997; Fischer, Jagels, & Hugli, 1999; Fukuoka & Hugli, 1988). Intracellularly, C3a plays an important role in activating the NLRP3 inflammasome in human monocytes (Asgari et al., 2013). Taken together, C3a promotes a proinflammatory environment. The C3 convertase also serves as the nucleus for the C5 convertase, when C3b molecules form a complex with C4b2b resulting in C4b2b3b, the C5 convertase of the LP and the CP, which cleaves C5 into the AT C5a and C5b (Figure 1) (Ekdahl et al., 2019). Similar to C3a and in concert with C3a, C5a can drive a proinflammatory environment through its strong chemotactic properties on neutrophils, monocytes, eosinophils, basophils, mast cell, and dendritic cell (DC) and its potency to activate such cells to release ROS, lysosomal enzymes, and proinflammatory cytokines such as IL‐1β, TNF‐α, IL‐6, and chemokines of the CC and CXC families (Figure 2). C5a also drives the activation and differentiation of T cells through DC maturation downstream of C5aR1 (Antoniou et al., 2020; Weaver et al., 2010). Also, C5a controls histamine‐induced increase in vasopermeability (Kordowski et al., 2019) and drives the production of metabolites of the arachidonic acid lipoxygenase and COX pathways resulting in increased production of LTB_4_ or PGE_2_ , both of which increase microvascular permeability (Karasu, Nilsson, Köhl, Lambris, & Huber‐Lang, 2019; Klos et al., 2009).

Importantly, such increased vascular permeability at the alveolar–capillary interface has been observed in infections with highly pathogenic respiratory viruses including H5N1 influenza, SARS‐CoV, and MERS‐CoV. It is associated with massive recruitment and activation of neutrophils resulting in excessive production of proinflammatory cytokines and chemokines including IL‐1β, IL‐6, IFN‐γ, IL‐8, CXCL10, and CCL2, eventually leading to the development of ALI/ARDS (Jiang et al., 2018, 2019). In experimental models as well as in patients infected with influenza (Ohta et al., 2011), SARS‐CoV (Gralinski et al., 2018), MERS‐CoV (Jiang et al., 2018), or SARS‐CoV‐2 (Gao et al., 2020; Magro et al., 2020), increased blood levels and/or lung deposits of complement activation products have been described.

Taken together, the picture emerges that highly pathogenic coronaviruses activate complement by the LP and CP. This activation drives the generation of huge amounts of the highly proinflammatory cleavage products C3a and C5a, when complement activation is not sufficiently controlled by complement regulator proteins (Figure 3). Further, TMA results in C3 and C5 cleavage by non‐canonical pathways through serine proteases located in the intracellular space of the vasculature that exert considerable enzyme activity (Figure 2) (Ekdahl et al., 2016). Below, we will discuss strategies to prevent the initiation of LP and CP, to attenuate convertase‐mediated amplification, and to inhibit the effector functions of C5a/C5aR1 axis activation.

### 5.2 Inhibition of LP and CP initiation

#### 5.2.1 Targeting the LP

Within the LP, the sensor molecules MBL, the ficolins 1–3, the collectins 10 and 11, or the serine proteases MASP‐1 and MASP‐2 could serve as potential targets. At this point, no strategies have been developed to target the sensor molecules. However, Omeros has developed the MASP‐2 targeting human antibody narsoplimab (OMS721) that is currently in phase III trials for HSCT‐TMA, IgA nephropathy, and atypical haemolytic uraemic syndrome (aHUS) and in a phase II trial for lupus and other renal diseases (Ricklin, Mastellos, Reis, & Lambris, 2018). For TMA, the FDA has granted narsoplimab a breakthrough designation in patients with persistent TMA as well as orphan drug designation for the inhibition of complement‐mediated TMAs and the treatment of HSCT‐TMA. Given that narsoplimab is already in clinical trials for diseases in which TMA is a critical disease driver, it might be worth considering this approach for severe cases of COVID‐19 infection (Figure 1).

In addition to MASP‐2, a C2‐blocking antibody (PRO‐02) has been developed by Prothix/Broteio to inhibit the formation of the C4b2b convertase of the LP and the CP and is being tested as a potential therapeutic approach for ischaemia‐reperfusion injury‐mediated disorders and autoimmune diseases (Borosss, Yildiz, Simons, Boon, & Hack, 2016).

#### 5.2.2 Targeting the CP

Potential targets specific for the CP are either the pattern recognition molecule C1q or the serine proteases C1r and C1s. Antibodies against C1q have been generated by Annexon, either as a complete monoclonal antibody (ANX005) or as a Fab fragment (ANX009), both of which have completed Phase 1b trials for Guillain–Barre syndrome or glaucoma showing full inhibition of CP activation. The FDA has granted ANX005 fast‐track and orphan drug designation for the treatment of Guillain–Barre syndrome. Antibodies are also available that are directed against C1s. Based on the mouse antibody TNT003 (Shi et al., 2014), the Sanofi subsidiary Bioverativ (a Sanofi company) has developed the humanized antibody sutimlimab (TNT009), which is currently tested in a phase III trial for cold agglutinin disease (CAD), a subtype of autoimmune haemolytic anaemia, and in a phase I trial for idiopathic thrombocytopenic purpura. In a small cohort of 10 patients suffering from CAD, sutimlimab was found to be safe, well‐tolerated, and rapidly stopped CP‐mediated haemolysis (Jager et al., 2019). In December 2019, Sanofi has reported first results from the phase III trial showing that sutimlimab inhibited haemolysis and improved anaemia and fatigue in CAD patients shortly after treatment (Mastellos, Ricklin, & Lambris, 2019). Finally, plasma protease C1 inhibitor (C1INH) controls the activity of C1s and has been on the market for more than 20 years for the treatment of hereditary angioedema. The problem with C1INH is that it targets not only C1s but also proteases of the coagulation, kinin, and fibrinolysis pathways (Levi, Cohn, & Zeerleder, 2019). Thus, for selective and tailored targeting of the CP, C1INH is not an option.

In summary, antibodies are available that specifically target the LP or the CP and are already in phase III trials. Given that both virus‐driven LP and adaptive immune response‐mediated CP activation by virus‐specific IgG and IgM antibodies will activate the complement system (Figure 1), it might not be sufficient to target only the LP.

#### 5.2.3 Targeting complement amplification at the level of C3

As aptly put by the Lambris Lab, C3 serves as the “Swiss army knife” of the complement proteins. C3b generated from C3 in response to CP, LP, or AP activation can amplify the initial complement activation by either pathway, when it forms the C3bB complex that can be cleaved by the serine protease FD, resulting in the self‐amplifying C3bBb convertase (Ricklin, Reis, Mastellos, Gros, & Lambris, 2016). This amplification loop will mediate the cleavage of many molecules of C3 when not appropriately controlled by complement regulator proteins (Figures 1 and 3). The available data suggest that such control by complement inhibitors of the regulator of complement activation (RCA) family is disturbed in patients developing ARDS and TMA following infection with highly pathogenic coronaviruses, in particular in African‐Americans (Figure 3). All of these RCA proteins harbour complement control protein domains. Some of them are membrane‐bound such as complement receptor 1 (CR1/CD35), MCP/CD46, and DAF/CD55, whereas others are found in the circulation (FH and C4 binding protein [C4BP]). Mechanistically, the RCA proteins either destabilize the C3 convertases or serve as cofactors for FI‐mediated degradation of C3b to iC3b and C3dg, which no longer contribute to the formation of the amplification loop. As implied by the name, DAF/CD55 accelerates the decay of the convertase, whereas CD46 mediates degradation of C3b. CR1, FH, and C4BP exert both functions.

Several compounds have been developed that inhibit the C3 convertase, either by targeting molecules that are critical for assembly (FB, FD, and MASP‐3) or by destabilization of the convertase complex and degradation of C3b (CR1 and FH). These compounds have recently been discussed in detail in two excellent reviews (Mastellos et al., 2019; Ricklin et al., 2018). Prima facie, FD is an attractive target in COVID‐19 infection, given that the proteolytic cleavage of FB by this serine protease is a crucial step to initiate the amplification loop. Also, plasma levels are relatively low, although high plasma turnover might pose a challenge. Thus, it is not surprising that small molecule inhibitors (Novartis, Achillion) and FD antibodies (Genentech) have been generated and tested in several clinical trials. Also, a MASP‐3‐specific antibody (OMS906) has been developed by Omeros to block the conversion of pro‐FD to FD (Dobo et al., 2016; Hayashi et al., 2019). However, several other serine proteases can cleave C3 including elastase from neutrophils or proteases of the coagulation, the kinin, and the fibrinolysis system. As the substrate specificity of these proteases for C3 is much lower than for their cognate substrates, the effects of such proteases under homeostatic conditions are probably minor. However, under pro‐thrombotic conditions such as TMA, when control systems are exhausted and intravascular protease inhibitor concentration is low, such proteases are likely to cleave C3 and drive AP activation (Ekdahl et al., 2019). In light of this consideration, blocking of systemic AP activation by specific targeting of FD or MASP‐3 seems difficult in patients suffering from ALI/ARDS with TMA or multi‐organ failure.

As an alternative to FD, FB‐targeting intervention has been developed by Ionis with Roche as a partner. They use a ligand‐conjugated antisense drug to reduce the production of FB, which is now in phase II trials for IgA nephropathy and age‐related macular degeneration (AMD). At this point, it is unclear how efficiently this drug would attenuate FB production in a severe systemic inflammatory disease state. We will only briefly mention molecules that destabilize the C3 convertase as clinical development of some of these molecules has either been discontinued (TP10 or TT30, extracellular variants of CR2) (Lazar et al., 2007; Risitano et al., 2012) or are still in preclinical development (mini‐FH, Amyndas) (Schmidt et al., 2013).

As an alternative approach to target C3, conversion to C3 convertase has been selected by the Lambris Lab. They identified a peptide from a phage library, compstatin, that prevents the binding of C3 to the assembled convertase independent of its origin (Sahu, Kay, & Lambris, 1996). Through several rounds of iteration, the affinity of this compound for C3 has been increased by more than 3 orders of magnitude as compared with the original compound, eventually leading to another peptide, CP40. This molecule served as a lead candidate for AMY‐101 (Amyndas), which now in phase II trials for periodontal disease and C3 glomerulopathy (Mastellos et al., 2019). This approach of direct C3 targeting in COVID‐19 is attractive, as it is aimed at blocking virus‐induced LP and CP activation as well as LP/CP‐driven activation of the amplification loop, at the bottleneck of all the pathways (Figure 1). However, C3 is one of the most abundant plasma proteins with a concentration in the range of 1.5 mg·ml^−1^. Thus, high amounts of inhibitor are required to efficiently reduce circulating C3. The high turnover of C3 under strongly inflammatory conditions adds to this problem. Finally, it remains to be determined whether compstatin derivatives would also prevent the cleavage of C3 by all circulating or cell‐derived serine proteases, as outlined above. Despite these challenges, direct C3 targeting appears an attractive target in severe infection with highly pathogenic coronaviruses. In support of this view, treatment of a patient suffering from severe ARDS in response to COVID‐19 infection with AMY‐101 resulted in a favourable disease course (Mastaglio et al., 2020). It will be important to further evaluate the benefit of C3 targeting with AMY‐101 in a large cohort of COVID‐19 patients.

The list of compounds that specifically target the interaction of the small cleavage product of C3, C3a with its C3aR, is short. The selective nonpeptide C3aR antagonist SB290157 was synthesized almost 20 years ago (Ames et al., 2001) and has been used, with varying success, in several preclinical models to target C3aR. However, no clinical development has been pursued. During the past 10 years, the Fairlie Lab has developed sophisticated approaches to design small molecule agonist and antagonists from different proteins including C3a (Reid et al., 2013). In this context, they have recently reported on the new compound JR14a, a very potent C3aR antagonist that is 100‐fold more potent than SB290157 (Rowley et al., 2020). This molecule awaits preclinical testing in animal models of inflammation.

### 5.3 Targeting the terminal pathway

#### 5.3.1 Lessons learned from targeting C5 in TA‐TMA

The generation of the terminal complement complex (C5b‐9, MAC) is initiated by the cleavage of C5 by the C5 convertase resulting in the generation of C5b and C5a. Once C5b is formed, C6 can bind to a labile binding site in C5b. Next, C7 can associate, followed by binding of the heterotrimeric C8α,β,γ which is critical for membrane insertion. The C5b8 complex serves as the receptor for C9 and drives its oligomerization, which is critical for membrane perforation, target cell lysis, and endothelial cell damage (Hadders et al., 2012). The MAC promotes inflammation by inducing the expression of adhesion molecules and the release of chemokines and PAF, which can ultimately lead to dysregulation of coagulation resulting in microvascular thrombosis. The ability of MAC to up‐regulate expression of leucocyte adhesion molecules on endothelial cells might also contribute to platelet localization and adhesion as well as increased leucocyte adhesion and subsequent cytokine and growth factor production (Dobrina et al., 2002).

Patients with TMA produce high amounts of the C5 cleavage products C5a and C5b and subsequently C5b‐9, due to enhanced LP, CP, or AP activation caused by defective complement regulation and/or excess activation (Figure 3). Elevated levels of circulating soluble C5b‐9 (sC5b‐9) can be found in the blood of patients with complement‐mediated TMAs. High concentration of sub‐lytic MAC in target cells may have a detrimental effect in a variety of tissues including the kidney, lung, and the CNS. Selective inhibition of C5 cleavage by C5‐specific antibodies is one of the options to inhibit formation of C5b‐9/MAC that has been successfully applied to clinical practice (Figure 1).

Eculizumab (Soliris, Alexion Pharmaceuticals) is a humanized murine monoclonal antibody against C5, which prevents C5 cleavage and the generation of C5b‐9/MAC by any of the three complement pathways. Eculizumab was first approved for the treatment of paroxysmal nocturnal haemoglobinuria (PNH). The efficacy and safety of eculizumab for treating aHUS were demonstrated in prospective clinical trials and this antibody has been adopted for therapy in high‐risk TA‐TMA patients (Legendre et al., 2013). Eculizumab (off‐label) has been successfully used in HSCT recipients with severe TA‐TMA and is one of the first complement blocking agents used to treat COVID‐19 patients.

Complement mediated TA‐TMA occurring in HSCT recipients very closely resembles histological and clinical TMA presentation in subjects with COVID‐19 suffering from a hyperinflammatory syndrome. The hyperinflammatory response in immunocompromised individuals with TA‐TMA is often triggered by viral pathogens such as the BK polyoma virus (Laskin et al., 2019), influenza/parainfluenza virus (Bitzan & Zieg, 2018), adenovirus (Yabe et al., 2005), or HHV‐6 (Belford et al., 2004). It is associated with very high systemic complement activation as measured by elevated blood sC5b‐9 and can lead to multi‐organ injury resembling clinical and autopsy reports in SARS‐CoV‐2. Untreated patients with complement mediated TA‐TMA have >80% mortality due to multi‐organ failure. Eculizumab treatment significantly improved survival as compared with untreated cohorts (66% vs. 17% 1 year post‐transplant survival) (Jodele et al., 2014, 2020). In HSCT recipients with TA‐TMA pre‐therapy, plasma sC5b‐9 was associated with the risk of dying from TMA. Plasma sC5b‐9 also correlated with increased eculizumab drug clearance and was incorporated as one variable in a pharmacokinetic/pharmacodynamic dosing algorithm for eculizumab in severely ill patients designed to achieve and maintain therapeutic eculizumab levels (>100 μg·ml^−1^) for prompt control of TA‐TMA (Jodele et al., 2016).

#### 5.3.2 C5 targeting with eculizumab in COVID‐19 patients

Due to the immediate need for clinical strategies to manage vigorous complement activation in SARS‐CoV‐2‐infected patients, we may adopt some of the available knowledge from complement‐mediated TA‐TMA in HSCT recipients. Eculizumab can be considered for the COVID‐19 population because of the significant amount of knowledge gained from using this drug in critically ill patients such as HSCT recipients with TA‐TMA, along with its acceptable toxicity profile and the lack of interference with T‐cell mediated anti‐viral responses (Jodele, Dandoy, et al., 2020). Importantly, a first case study which applied eculizumab to COVID‐19 patients suffering from ARDS or severe pneumonia resulted in successful recovery of all patients with reduction in inflammation (Diurno et al., 2020). Four subjects with confirmed severe COVID‐19‐associated pneumonia with oxygen requirement and radiological evidence of bilateral pneumonia were offered eculizumab. Despite presenting with a rapidly worsening respiratory function, all patients showed a marked clinical improvement within the first 48 h after the first eculizumab dose with a median therapy time of 12.8 days to disease resolution. These first encouraging data warrant further evaluation in a larger cohort of COVID‐19 patients. Last year, Alexion launched ravulizumab, a variant of eculizumab, with longer plasma residence than eculizumab, allowing a less frequent dosing interval of 8 weeks (Kulasekararaj et al., 2019; Lee et al., 2019). Recently, the company announced to conduct a phase III, open‐label, randomized and controlled study to determine the safety and efficacy of ravulizumab in COVID‐19 patients with severe pneumonia, ALI, or ARDS (Alexion, 2020).

Monitoring blood sC5b‐9 can potentially serve as a helpful surrogate marker for enhanced C5 production in COVID‐19 patients, as the turnover of C5 will determine the clearance of eculizumab. In a high inflammatory state, as can be seen in severely ill HSCT recipients with TA‐TMA or COVID‐19 patients, there is an acute phase response of the liver with massive C5 production and additional C5 production by activated circulating inflammatory cells and injured endothelial cells. Under such conditions, there are more target C5 molecules generated, and more eculizumab is required as it forms immune complexes with the increased number of C5 molecules. Eculizumab serum concentration, sC5b‐9, and CH50 monitoring tests are clinically available that can be adopted for pharmacokinetic/pharmacodynamic‐guided eculizumab dosing in COVID‐19 patients, as already described in HSCT populations (Jodele, Fukuda, et al., 2016). All patients receiving complement blockers should additionally receive antimicrobial prophylaxis appropriate for the prevention of meningococcal infection, as the available meningococcal vaccine does not provide adequate protection (Bouts, Monnens, Davin, Struijk, & Spanjaard, 2011; Struijk et al., 2013). Complement blockade using eculizumab with appropriate antimicrobial prophylaxis was shown to be safe in immunocompromised HSCT recipients (Jodele et al., 2016).

#### 5.3.3 Alternative strategies to target C5

Genetic C5 variants have been identified that result in poor binding of eculizumab and treatment failure in patients suffering from PNH (Nishimura et al., 2014). In addition to eculizumab, several other C5‐targeting antibodies have been developed by Roche, Novartis, and Regeneron. For example, Roche and Chugai developed SKY/RO7112689, which is effective in patients with the C5 variant p.Arg885His and exerts long‐lasting inhibition of C5 (Fukuzawa et al., 2017). Also, Novartis (LFG316) and Regeneron (pozelimab/REGN3918) generated anti‐C5 antibodies that are currently in clinical development.

The recombinant small protein nomacopan (Coversin, Akari Therapeutics) from the *Ornithodros moubata* tick is another molecule that targets C5 and prevents the release of C5a and formation of C5b–9, although in a different way from eculizumab (Jore et al., 2016). In addition to C5, it also targets LTB_4_. Nomacopan was shown to disrupt cell trafficking (in particular that of neutrophils) (Figure 2) and the release of proinflammatory cytokines in several experimental models of ARDS and sepsis including those induced by viral infections like influenza H1N1 (Garcia et al., 2013). It is likely that C5a and LTB_4_ together account for many of the proinflammatory effects associated with pulmonary inflammation and TMA (Figure 2) as observed in COVID‐19 patients with severe courses. Nomacopan is administered as a continuous subcutaneous infusion, which will potentially provide continuous complement blockade in the circulation. It demonstrated a promising complement modulating response in TA‐TMA and is now being examined in phase III clinical trials (Goodship et al., 2017).

Although terminal complement blockade significantly improved TA‐TMA therapy, a more tailored approach targeting C5a or C5aR1 might be sufficient to cope with the deleterious, proinflammatory effects of overactivated complement.

#### 5.3.4 Blockade of the C5a/C5aR1 axis

C5a is generated in response to C5 cleavage by canonical and non‐canonical complement activation. It exerts many of its proinflammatory properties through engagement of its cognate GPCR C5aR1. It also binds to C5aR2, which is uncoupled from G‐proteins (Karasu et al., 2019; Klos et al., 2009). At this point, most strategies to target C5a‐mediated effects focus on C5aR1 (Figure 1). The most advanced approach uses a small molecule inhibitor of C5aR1, avacopan (CCX168), developed by ChemoCentryx that is orally available. In a phase II trial for ANCA‐associated vasculitis, efficacy, safety and a steroid‐sparing effect (Jayne et al., 2017) has been demonstrated for avacopan. These findings were recently confirmed by first data released from a phase III trial, which even suggested superiority to standard glucocorticoid therapy (ChemoCentryx, 2019). Innate Pharma has developed the fully human anti‐C5aR antibody avdoralimab (IPH‐5401), which is currently tested in patients with advanced solid tumours in a phase I trial together with the PD‐L1 antibody durvalumab (AstraZeneca). Additional C5aR1 antagonists have been developed, which are still in the preclinical stage including an allosteric inhibitor of C5aR1 (Dompe) or the cyclic peptide ALS‐205 (Alsonex) based on PMX‐53, a non‐competitive inhibitor of C5aR1, which has been successfully used in several animal models of inflammatory diseases to target C5aR1 (Hawksworth, Li, Coulthard, Wolvetang, & Woodruff, 2017). Finally, the C5a mutein A8^Δ71–73^ has been developed, primarily selected from a phage‐display library, that simultaneously targets C5aR1 and C5aR2 (Heller et al., 1999). The antagonistic properties rely on an amino acid replacement at position 69 of C5a with a positively charged amino acid (Otto et al., 2004). This molecule was superior to isolated C5aR1 targeting, in a preclinical model of sepsis (Rittirsch et al., 2008).

As an alternative approach, InflaRx has developed the monoclonal antibody IFX‐1 that specifically targets hC5a (Figure 1). This antibody, which has been licensed to Staidson Biopharmaceutics (BDB‐001), is currently used in a multicentre, randomized double‐blind placebo‐controlled trial in mild COVID‐19 patients and an open‐label two‐cohort clinical trial in patients with severe and critical COVID‐19. First results were recently released showing a promising curative effect in two severe COVID‐19 patients with moderate ARDS or pneumonia (Gao et al., 2020). InflaRx has also initiated a phase II study in Europe with IFX‐1 in COVID‐19 patients with severely progressed pneumonia (InflaRx, 2020).

The available preclinical data and the few clinical data point towards a key role for C5a in complement‐driven ARDS and TMA development in response to highly pathogenic coronavirus infection. The strong differences between C5a serum levels of COVID‐19 patients with moderate and severe disease (Gao et al., 2020) indicate that longitudinal monitoring of C5a serum levels in patients with moderate disease might help to identify and stratify patients at risk of developing severe lung injury and TMA. Targeting C5a or C5aR1 might be more effective than targeting C5, as it is a more selective approach that leaves the formation of the MAC intact, which is critical to combat infections with encapsulated bacteria including *Neisseria meningitidis.* Also, C3‐fragment‐mediated opsonization is still possible. Further, the C5a/C5aR1 axis interacts with and amplifies the responses of other innate immune receptors. For example, C5aR1 sets the threshold for IgG Fc receptor (FcγR)‐mediated immune cell activation, as its activation up‐regulates the expression of activating FcγRs and down‐regulates the expression of inhibitory FcγRIIB (Karsten & Köhl, 2012). Of note, a significant association has been described between an SNP in FcγRIIA and the severity of SARS‐CoV infection (Yuan et al., 2005). Thus, C5aR targeting might also reduce virus‐IgG‐driven immune cell activation by activating FcγRs.

## 6 COMPLEMENT INTERACTION WITH OTHER INFLAMMATORY PATHWAYS AND POTENTIAL CONCURRENT INTERVENTIONS

Complement system dysregulation is one of the major pathways leading to endothelial injury. While complement blockade improves TMA, not all patients respond to therapy, prompting a search for additional targetable pathways of endothelial injury. Emerging data from COVID‐19 patients demonstrate the interplay of multiple inflammatory pathways. Thus, novel personalized strategies including combination therapies might be required to effectively fight the hyperinflammatory storm (Figure 2) (Barnes et al., 2020; Giamarellos‐Bourboulis et al., 2020; Gloude et al., 2017; Zhao, 2020). In support of this view, proteomic and metabolomic profiling of sera from healthy controls and patients with non‐severe and severe COVID‐19 infection identified changes in complement pathways in concert with changes in platelet degranulation and macrophage function as the main variables to predict progression to severe COVID‐19 disease (Shen et al., 2020). Also, the authors found a strong increase in CRP in severely ill COVID‐19 patients, which is a strong non‐canonical activator of complement by the CP (Biro et al., 2007). In another systems approach, activation of the complement system, the kinin–kallikrein pathways and IL‐6 were identified as the main pathways responsible for the dysregulation of inflammation in patients with severe COVID‐19 infection (Van de Veerdonk et al., 2020). All of these pathways have formerly been associated with the development of TMA.

Recent data in HSCT recipients with TA‐TMA suggest a key relationship between complement activation and increased IFN signalling, NETs, and chemokines/cytokines such as IL‐8 and IL‐6 forming an “IFN‐complement loop” that can perpetuate endothelial injury and TMA. Recent RNAseq data in HSCT recipients with TMA showed that IFNs promote expression of complement genes, such as C1Q, which initiates the classical complement pathway and ultimately leads to formation of the MAC/C5b‐9 and endothelial injury presenting as TMA (Jodele et al., 2020). Intracellular complement C5 production, cleavage into C5a, and intracellular C5aR1 activation in response to T cell activation result in NLRP3 inflammasome activation, Th1 differentiation, and production of IFN‐γ which could fuel the inflammatory scenario and sustain endothelial cell damage (Arbore et al., 2016).

Injured endothelial cells release IL‐8, causing neutrophil activation and formation of NETs. In response, NET formation promotes complement system activation via the AP and FP (properdin) binding (Yuen et al., 2016). NET production can be further stimulated by IFN‐γ (Gloude et al., 2017). IFN‐α and IFN‐β proteins increase differentiation of B cells into plasma cells that can produce anti‐FH antibodies, preventing inhibition of the AP. NETs can activate plasmacytoid DCs to produce high levels of IFN‐α that can directly activate complement via C5b‐9, resulting in vascular endothelial injury (Umemura et al., 2015).

Therapeutic administration of IFNs has been shown to cause TMA (Garcia‐Romo et al., 2011). Viral pathogens that can trigger high IFN‐γ production may also lead to development of complement‐mediated TMA (Zareei et al., 2019). In addition, viruses can directly injure endothelial cells and promote release of IFN‐γ (An, Saenz Robles, Duray, Cantalupo, & Pipas, 2019). Inflammatory chemokines/cytokines, such as IL‐6, CXCL8/IL‐8, and IFN‐γ, are also released from circulating activated T cells, NK cells, monocytes, and tissue macrophages as a response to viral infection, again contributing to TMA development.

A better understanding of the “IFN‐complement loop” provides new opportunities to combine therapies that might be used as personalized treatment options for defined patient cohorts. One clinical example is haemophagocytic lymphohistiocytosis (HLH), a rare clinical syndrome of excessive immune activation, characterized by signs and symptoms of extreme inflammation, driven mainly by IFN‐γ and other proinflammatory cytokines with good response to emapalumab (Lounder, Bin, de Min, & Jordan, 2019; Vallurupalli & Berliner, 2019), a human monoclonal antibody to IFN‐γ. It is approved for treatment of severe HLH. Patients with HLH, who simultaneously present with complement‐mediated TMA, have high incidence of multi‐organ injury and poor outcomes. Case series in children suggest that combined inhibition of IFN‐γ and the terminal complement pathway in TMA might provide faster disease control and recovery from organ injury than targeting either IFN‐γ or C5 (Gloude et al., 2020). Given that patients with severe COVID‐19 infection show massive activation of several inflammatory pathways, monitoring complement (C3a, C5a, and sC5b‐9) and IFN‐γ pathway activation (CXCL9) as well as IL‐6 levels in the circulation in the course of COVID‐19 infection could have immediate clinical implications. Algorithms might be developed on the basis of these pathway activation patterns for patient risk stratification and targeted interventions using currently available drugs to halt COVID‐19 progression to multi‐organ failure and improve outcome.

## 7 CONCLUSION AND FUTURE PERSPECTIVE

In summary, the available data strongly support a model in which complement activation in the lung and in other organs is a critical host mediator of SARS‐CoV‐2‐induced development of atypical ARDS and TMA. We would like to propose a model in which strong complement activation by the LP and/or the CP occurs in patients suffering from atypical ARDS/TMA ,resulting in massive generation of C5a. Polymorphisms in exon 1 and/or the promoter region of MBL or in complement regulators may define the extent of complement activation, in particular in African‐Americans. Alternatively, and not mutually exclusive, the extent of virus‐specific neutralizing IgG Abs generated after the first week of infection may determine the magnitude of complement activation. Importantly, C5a controls the threshold of IgG Fc receptor expression as an important mechanism of IgG‐mediated innate immune cell activation (Karsten & Köhl, 2012). Complement activation occurs primarily in the lower airways but will result in the release of C5a into the circulation. Such C5a recruits and activates proinflammatory immune cells as a key mechanism that drives the “cytokine and chemokine storm” associated with fatal lung injury and TMA development (Figure 2). Thus, targeting C5, C5a, or its primary receptor, C5aR1, should be considered to alleviate the proinflammatory effects, reduce lung pathology, and increase the survival of COVID‐19 patients.

### 7.1 Nomenclature of targets and ligands

Key protein targets and ligands in this article are hyperlinked to corresponding entries in http://www.guidetopharmacology.org, the common portal for data from the IUPHAR/BPS Guide to PHARMACOLOGY (Harding et al., 2018), and are permanently archived in the Concise Guide to PHARMACOLOGY 2019/20 (Alexander, Christopoulos et al., 2019; Alexander, Fabbro et al., 2019).

## CONFLICT OF INTEREST

J.K. has no conflict of interest to declare. S.J. submitted a US patent application entitled: Compositions and methods for treating TA‐TMA. Further, she conducted an NIH‐funded study with the study drug provided by Alexion Pharmaceuticals at no charge to study subjects. Finally, she received travel support from Omeros Corporation.
